# Serial serum creatinine, SDMA and urinary acute kidney injury biomarker measurements in dogs envenomated by the European adder (*Vipera berus*)

**DOI:** 10.1186/s12917-021-02851-8

**Published:** 2021-04-12

**Authors:** Hannah J. Harjen, Tove V. Nicolaysen, Tale Negard, Hege Lund, Bente K. Sævik, Kristin P. Anfinsen, Elena R. Moldal, Karin E. Zimmer, Runa Rørtveit

**Affiliations:** 1 Faculty of Veterinary Medicine, Department of Companion Animal Clinical Sciences, Norwegian University of Life Sciences, Oslo, Norway; 2 Faculty of Veterinary Medicine, Department of Preclinical sciences and Pathology, Norwegian University of Life Sciences, Oslo, Norway; 3 Anicura Jeløy Dyresykehus, Moss, Norway

**Keywords:** Acute kidney injury, Adder, Viper, Snakebite, Biomarker, Renal

## Abstract

**Background:**

Acute kidney injury (AKI) is associated with high morbidity and mortality in dogs, but diagnosis may be impaired due the insensitivity of routine renal function biomarkers to detect earlier or milder forms of injury. Snake envenomation is one of several causes of AKI in dogs and humans. Dogs are commonly envenomated by the European adder (*Vipera berus*) between April and October each year, but few studies exist examining serial serum creatinine (sCr) and symmetric dimethylarginine (SDMA) measurements and AKI biomarkers in these dogs. Novel urinary biomarkers could improve clinical outcome by allowing earlier diagnosis of and intervention in AKI. The aim of this study was to assess the presence of AKI in dogs envenomated by *V. berus* at 12, 24 and 36 h after bite, as well as 14 days later, using sCr, SDMA and a panel of urinary AKI biomarkers normalised to urine creatinine (uCr), compared to a group of healthy control dogs.

**Results:**

Thirty-five envenomated dogs and 35 control dogs were included. Serum creatinine did not exceed the upper reference limit at any time point in any dog after envenomation. Serum SDMA did not exceed 0.89 μmol/L in any dog. Compared to controls, urinary albumin/uCr, neutrophil gelatinase-associated lipocalin/uCr and monocyte chemotactic protein-1/uCr were significantly elevated 12 h (*P* <  0.0001, *P* <  0.0001, *P* = 0.01), 24 h (*P* <  0.001, *P* <  0.001, *P* = 0.002) and 36 h (*P* <  0.001, *P* <  0.001, *P* = 0.0008) after bite. Osteopontin/uCr was higher 24 and 36 h after bite (*P* < 0.0001), kidney injury molecule-1/uCr, interleukin-8/uCr and γ- glutamyl transferase/uCr were significantly higher 36 h after bite (*P* = 0.003, *P* = 0.0005, *P* = 0.001). Urinary cystatin C/uCr was not significantly different to controls at any timepoint. Biomarker/uCr ratios were not significantly different 14 days after envenomation compared to controls.

**Conclusion:**

Urinary biomarker/Cr ratios are indicative of mild transient, non-azotaemic AKI in dogs envenomated by *V. berus.*

**Supplementary Information:**

The online version contains supplementary material available at 10.1186/s12917-021-02851-8.

## Background

Acute kidney injury (AKI), historically defined as an abrupt reduction in renal function [1], carries a substantial risk of mortality in dogs [2, 3]. Acute kidney injury represents a wide spectrum of disease, from subclinical injury to overt kidney dysfunction, with associated clinical signs including anorexia, vomiting, diarrhea, lethargy, polyuria, oliguria, anuria and polydipsia [4]. Causes of AKI are varied and include ischemia, infection, drugs such as non-steroidal anti-inflammatory drugs and aminoglycosides, and toxins including snake venom [5, 6].

Snakebite-related AKI is described in humans and dogs [5–7]. Envenomation by the European adder *(Vipera berus)* is a common occurrence in dogs in Scandinavia during April to October. Studies to date have failed to show elevations in renal function markers such as serum creatinine (sCr) after *V.berus* envenomation, but measurements of urinary markers of AKI and histopathological findings are indicative of tubular injury in these dogs [5, 8, 9]. These studies are however limited, and AKI in dogs envenomated by *V. berus* therefore warrants further investigation. Snakebitten dogs often present for treatment within a short time of insult, prior to clinical signs referable to acute kidney dysfunction. Thus, *V.berus* envenomated dogs could also represent a model for studying kidney injury biomarkers in the early phase of AKI.

Serum creatinine, the surrogate marker of glomerular filtration rate (GFR) routinely used to assess kidney function in dogs, is insensitive for diagnosing early or mild dysfunction [10, 11]. Serum symmetric dimethylarginine (SDMA) is reportedly a more sensitive and specific marker for GFR than sCr in dogs [11]. The use of SDMA in diagnosing early or mild forms of AKI is not fully established. Furthermore, kidney damage can be present without subsequent loss of function [12], thus limiting the use of such functional biomarkers in AKI diagnosis. Emergency treatment of envenomated dogs often involves fluid therapy, which can further hinder the diagnostic use of serum biomarkers for AKI due to haemodilution. Despite its limitations, sCr forms the basis of current AKI grading systems. Diagnosis of AKI is therefore largely based on identifying dysfunction and not necessarily damage, meaning that early or milder stages of kidney injury may go undetected.

Various AKI grading systems based on sCr concentrations and urine output are used in human medicine [1, 13, 14] and have been adapted for veterinary patients [3, 15, 16]. AKI can progress to chronic kidney disease [17–19],and higher grades are associated with increased mortality in both humans and dogs [3, 15, 20, 21]. Earlier diagnosis of AKI could allow clinicians to intervene and prevent further progression of kidney disease. It would therefore be of benefit to develop more sensitive methods of diagnosing AKI, using biomarkers able to detect injury in the absence of altered GFR as measured by sCr. This would aid diagnosis of mild or early stage AKI which, although subclinical, might be of importance [22].

A variety of low and high molecular weight (LMW and HMW) urinary proteins have been proposed as more sensitive and location specific (glomerular versus tubular) biomarkers of AKI than sCr. Urinary albumin can be an indicator of early kidney disease in dogs and AKI in humans [23], but its use can be limited by lack of specificity [24]. Urinary alkaline phosphatase (uALP) and γ- glutamyl transferase (uGGT) are proximal tubule brush border enzymes previously shown to be elevated in dogs with histological evidence of AKI [25, 26]. Kidney injury molecule-1 (KIM-1), neutrophil gelatinase-associated lipocalin (NGAL), interleukin 8 (IL-8), osteopontin (OPN) and monocyte chemotactic protein-1 (MCP 1) are upregulated upon renal tubular injury with increased urinary concentrations found in AKI of various etiologies in both humans and dogs [27–32]. Urinary Cystatin C (uCysC), a freely filtered LMW protein metabolised by renal tubular cells, is reportedly elevated in dogs with proximal tubular injury [33].

The aim of this study was to investigate AKI in *V. berus* envenomated dogs using sCr and a panel of urinary biomarkers for glomerular and tubular injury in the initial 48 h after envenomation and 14 days later, compared to a group of healthy controls. A secondary aim was to investigate associations between severity of clinical signs of envenomation and AKI biomarker concentrations. We hypothesized that dogs sustain kidney tubular injury following *V. berus* envenomation and that biomarker concentrations correlate with clinical severity of envenomation.

## Results

### Control dogs

The control group comprised various breeds (additional file 1). Eighteen (51%) were female, of which 4 were neutered, and 17 (49%) were male, of which 1 was neutered. Median age was 5 years (range 8 months – 13 years). Median weight was 24 kg (range 5.2–50 kg).

### Envenomated dogs

Thirty-five dogs of various breeds (additional file 1) envenomated by *V. berus* were included in the final study group. Twenty-two cases presented to the small animal hospital at the Norwegian University of Life Sciences (NMBU), three cases to Anicura Dyresykehus Oslo (ADO), six cases to Evidensia Oslo Dyresykehus (EOD) and four cases to Anicura Jeløy Dyresykehus (AJD). The snake or fang marks were witnessed in 25 dogs. Diagnosis was based on a history and clinical signs consistent with envenomation in the remaining dogs. Twenty (57%) dogs were female, of which 3 were neutered, and 15 (42.8%) were male, of which 3 were neutered. Median age was 4.5 years (range 7 months-18 years). Median weight was 19 kg (range 5.5–46.5 kg).

Twenty-four dogs were bitten in the head, 10 in a limb, and one on the sternum. Median time from bite to initial presentation was 1.6 h (range 0.5–9 h). Due to transfer of veterinary care, five dogs presented more than 8 h after bite and thus lack a T1 blood sample. Two dogs had been diagnosed with a *V. berus* bite six and five years previously.

### Treatment

All envenomated dogs were treated with crystalloid fluid therapy IV during hospitalisation (ringer-acetate (*n* = 34) or NaCl on day one followed by ringer-acetate on day two (*n* = 1)). Median fluid rate was 4 ml/kg/hour (range 2.7–6.3). Twenty-seven dogs received equine F(ab’)2 antivenom IV (Viper Venom Antitoxin, SIS Biomed®, Warsaw, Poland). Median time from bite to antivenom treatment was 4 h (range 0.5–24 h). Analgesics included buprenorphine (Vetergesic vet®, Ceva Santé Animale, France) at a dose of 0.01–0.02 mg/kg IV, IM or SC q8 hours (*n* = 6) or methadone (Metadon, Norges Apotek, Norway) at a dose of 0.1–0.2 mg/kg IV or SC q 4 h (*n* = 22). Six dogs received a combination of methadone and buprenorphine. One dog received a combination of methadone and transdermal fentanyl at a dose of 4 μg/kg/h (Durogesic®, Janssen-Cilag AS). Lidocaine (Xylocain®, Aspen Pharma trading Ltd., Ireland) was administered at dose of 1 mg/kg to one dog within one hour of presentation due to a ventricular arrhythmia. Three dogs were treated with maropitant at a dose of 1 mg/kg IV due to hypersalivation or vomiting. One dog was administered furosemide (2 mg/kg IV) prior to T3 due to perceived oliguria. Other treatments included ampicillin (n = 1), streptomycin and penicillin (n = 1), clindamycin between T4 and T5 (n = 1), amoxicillin between T4 and T5 (n = 1) dexamethasone (n = 1), prednisolone (n = 1), pimobendan (n = 1) and levothyroxine (n = 1).

### Blood pressure

Blood pressure was measured in 23/35 envenomated dogs. Overall median systolic arterial pressure (SAP) was 135 mmHg (range 104–196 mmHg), median diastolic arterial pressure (DAP) was 76 mmHg (range 48–154 mmHg) and median mean arterial pressure (MAP) was 97 mmHg (range 70–171 mmHg). No dogs were considered hypotensive (SAP < 90 mmHg or MAP < 60 mmHg and clinical signs of hypotension). Nine dogs had SAP values > 150 mmHg at one timepoint or more, of which four dogs had SAP values over 160 mmHg. Two of those four dogs had SAP values persistently > 160 mmHg, whereas two had SAP > 160 only at presentation, (including the individual with SAP of 196 mmHg).

### Snakebite severity score (SSS)

Median SSS at presentation was 4/16 (range 0–12) and 4/16 (range 0–10) during T2-T4. At T5 all dogs had a score of 0, except for two dogs with scores of 1 and 2 respectively.

### Urinalysis

Median USG in the envenomated dogs was 1.022 (range 1.006–1.058) at T2-T4, and 1.036 (range 1.005–1.053) at T5. Median urine pH in the samples analysed from envenomated dogs was 7 (range 5–9). Of the envenomated dogs, five had a positive blood dipstick in the absence of erythrocytes on urine microscopy, possibly suggesting the presence of haemoglobinuria, and 13 had varying degrees of haematuria (median 50 red blood cells (RBC) /μL (range 10–250), median 22.5 RBC/ HPF (range 3–100). Macroscopic haematuria was not observed. One dog had myoglobinuria at T2, diagnosed by brown urine in the absence of haemolysed serum or erythrocytes on microscopy. The same dog had pigmenturia at T4 with concurrent haemolysis, thus haemoglobinuria, myoglobinuria or both were present at that single timepoint. Cellular (1/HPF) and granular casts (1–3/HPF) were observed in one and two dogs respectively.

Median USG and urine pH were 1.045 (range 1.018–1.060) and 6.5 (range 5–8) respectively in the control dogs.

### Biomarker analysis

Urinary ALP was deemed low and statistical analysis not performed due to 99% of samples having values under the limit of quantification (LOQ) for the assay. Urinary albumin, uIL-8, uMCP-1, uNGAL and uKIM-1 had 77.5, 68, 31, 20 and 3% of values under the LOQ for the assay, respectively. Values under the LOQ for these analytes were imputed using the lower LOQ (LLOQ) for the assay. For the remaining biomarkers, all values were over the LLOQ.

Serum creatinine, SDMA and urinary biomarker/uCr values are presented by timepoint for envenomated dogs and controls in Table 1. Serum creatinine did not exceed the upper reference limit at any time point in envenomated dogs. Two envenomated dogs did however have an increase in sCr of 26.5 μmol/L between T2 and T4 and T2 and T3 respectively. Symmetric dimethylarginine did not exceed the upper reference limit (> 0.74 μmol/L) in envenomated dogs at T1-T4. At T5 two dogs had SDMA concentrations of 0.79 and 0.84 μmol/L with corresponding USGs of 1.036 and 1.041 respectively. These were different dogs to those with increases in sCr.

**Table 1:** Median (range) biomarker values by timepoint after bite in envenomated dogs and healthy controls

	Control	T1 (presentation)	T2 (12 h)	T3 (24 h)	T4 (36 h)	T5 (14 days)
sCr (μmol/L)	88.4 (44.2–150.3)	70.7 (53–106)	61.88 (53–97.2)	66.3 (44.2–88.4)	70.7 (44.2–97.2)	79.6 (53–123.8)
SDMA (μmol/L)	0.54 (0.35–0.74)	0.5 (0.35–0.64)	0.47 (0.25–0.64)	0.45 (0.25–0.64)	0.4 (0.25–0.69)	0.54 (0.3–0.84)
uKIM-1/uCr (ng/mg)	0.13 (0.013–0.36)		0.13 (0.001–0.2)	0.15 (0.002–0.32)	0.20 (0.008–0.35)	0.14 (0.03–0.25)
uOPN/uCr (ng/mg)	1.86 (0.13–4.2)		2.54 (0.08–7.96)	5.22 (0.22–24.87)	6.34 (0.5–31.86)	1.88 (0.26–15.46)
uCysC/uCr (ng/mg)	0.95 (0.16–3.74)		0.66 (0.22–1.43)	0.57 (0.17–2.94)	0.56 (0.24–1.78)	0.58 (0.17–2.39)
uNGAL/uCr (ng/mg)	0.21 (0.02–2.94)		2.8 (0.1–15.78)	2.1 (0.07–3.02)	2.62 (0.09–21.66)	0.28 (0.03–23.3)
uMCP-1/uCr (ng/mg)	0.46 (0.05–1.97)		0.8 (0.15–5.44)	0.78 (0.23–7.24)	0.88 (0.27–5.58)	0.6 (0.09–6.14)
uGGT/uCr (U/g)	15.4 (0.95–75.26)		30.14 (3.96–83.44)	21.6 (0–85.65)	28.52 (9–120.5)	18.27 (0–48.83)
uALB/uCr mg/g	0.005 (0.002–6) 32/34 (94.1%) < LOQ*		0.019 (0.03–289) 9/16 (56%) < LOQ	0.036 (0.004–116) 16/25 (64%) <LOQ	0.025 (0.01–175) 17/22 (77%) < LOQ	0.007 (0.004–124) 16/19 (84%) < LOQ
uIL-8/uCr (ng/mg)	0.06 (0.02–0.9) 18/34 (52.9%) < LOQ**		0.08 (0.02–0.29) 10/16 (62.5%) < LOQ	0.14 (0.03–1.62) 21/24 (87.5%) < LOQ	0.14 (0.05–0.79) 18/22 (81.8%) < LOQ	0.07 (0.03–3.37) 14/20 (70%) < LOQ
uALP/uCr (U/g)	100% < LOQ***		100% < LOQ	100% < LOQ	96% < LOQ	100% < LOQ

* Limit of quantification (LOQ) = 9.6 ng/mL, **LOQ = 0.07 ng/mL, *** LOQ = 10 U/L

#### Comparison of Envenomated dogs to control dogs

**Fig. 1:**
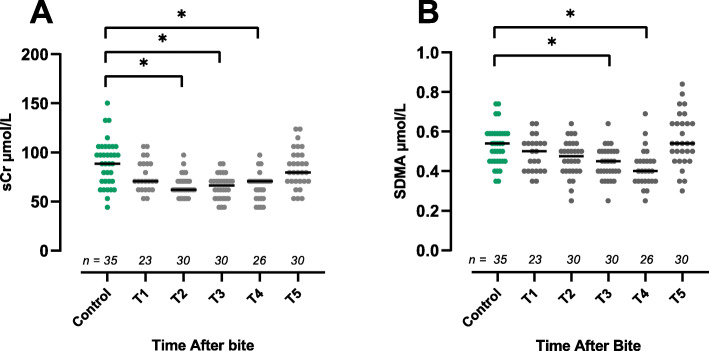
Serum creatinine (sCr) (**a**) and SDMA (**b**) concentrations for control dogs and each timepoint after bite for envenomated dogs. T1 = 0–7.5 h, T2 = 10–14 h, T3 = 22–26 h, T4 = 34–38 h, T5 = 10–23 days. Bar denotes median. * indicates *P* < 0.0001

**Fig. 2:**
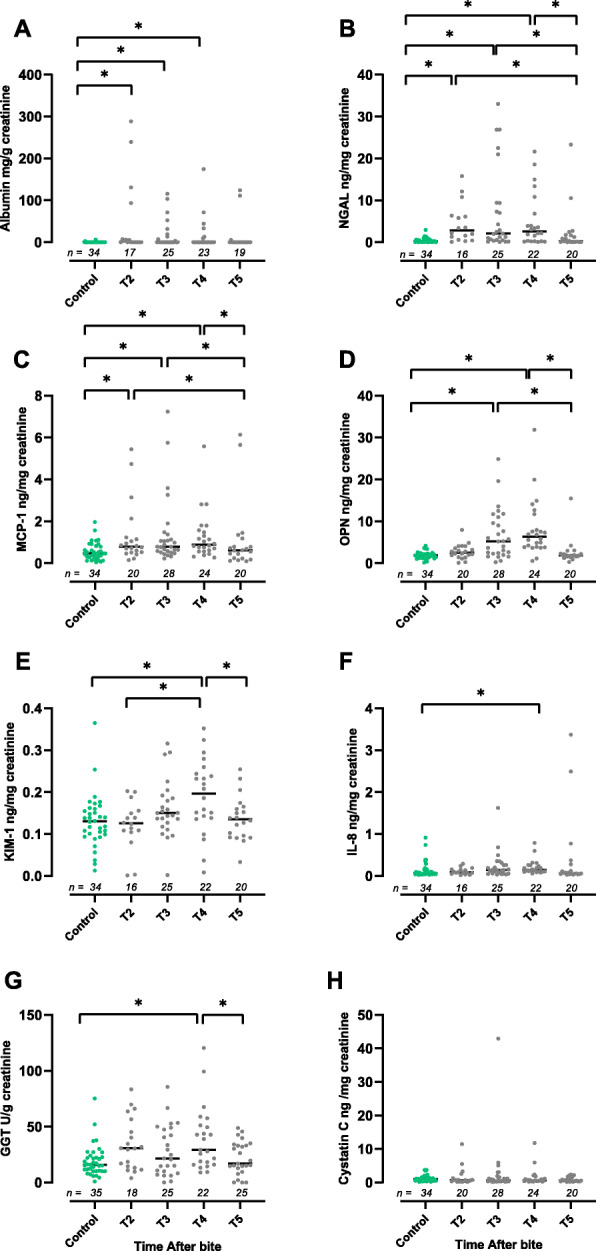
Urinary biomarkers NGAL, KIM-1, OPN, CysC, MCP-1, IL-8, albumin and GGT normalised to urine creatinine in control dogs and each timepoint after bite for envenomated dogs. T = 0–7.5 h, T2 = 10–14 h, T3 = 22–26 h, T4 = 34–38 h, T5 = 10–23 days. Bar denotes median, * indicates *P* < 0.0125 for comparisons with the control group and *P* < 0.008 for timepoint comparisons for envenomated dogs. For Cystatin C, IL-8 and albumin, comparisons of time points within the envenomated group were not made due to poor fitting of the regression model

Serum creatinine (Fig. 1 A) was significantly lower in envenomated dogs at T2, T3 and T4 compared to controls (*P* < 0.0001). Symmetric dimethylarginine (Fig. 1 B) was significantly lower at T3 (*P* = 0.0006) and T4 (P < 0.0001), compared to controls. No significant difference in sCr And SDMA was found at T1 or T5 compared to controls. Urinary Alb/uCr and NGAL/uCr were significantly higher compared to the control group at T2, T3 and T4 (*P* < 0.0001, Fig. 2 A and B). Urinary MCP-1 /uCr was significantly higher at T2 (*P* = 0.01), T3 (*P* = 0.002) and T4 (*P* = 0.0008, Fig. 2 C) compared to controls. Urinary OPN/uCr was significantly higher at T3 and T4 compared to controls (*P* < 0.0001, Fig. 2 D). Urinary KIM-1/uCr, uIL-8/uCr and uGGT/uCr were significantly higher at T4 compared to controls (P = 0.002 (Fig. 2 E*)*, P = 0.002, (Fig. 2 F*)* and *P* = 0.003, (Fig. 2 G*)* respectively). Urinary CysC/uCr was not significantly higher at any timepoint compared to control dogs *(*Fig. 2 H*).*

Eight of the envenomated dogs had microalbuminuria at one time point or more. Two dogs had microalbuminuria at T5.

#### Comparison of time points for envenomated dogs

Urinary KIM-1/uCr was significantly higher at T4 compared to T5 (*P* = 0.012, Fig. 2 E), uOPN/uCr was higher at T3 and T4 compared to T5 (*P* < 0.0001) and at T3 and T4 compared to T2 (*P* = 0.0003 and P < 0.0001 respectively, Fig. 2 D), MCP-1/Cr was higher at T2, T3 and T4 compared to T5 (*P* = 0.0012, *p* < 0.0001 and *P* = 0.0002 respectively, Fig. 2), uNGAL/uCr was higher at T2, T3 and T4 compared to T5 (P = 0.0002, P < 0.0001 and P = 0.0002 respectively, Fig. 2 B) and uGGT/uCr was significantly higher at T4 compared to T5 (P < 0.0001, Fig. 2 G). Effects of explanatory variables within the model are reported in Table 2.

**Table 2:** *P* values for mixed model analysis for the effect of explanatory variables on urinary biomarker/creatinine ratios

Explanatory Variable	Response Variable				
	uKIM-1/uCr ng/mg	uOPN/uCr ng/mg	uMCP-1/uCr ng/mg	uNGAL/uCr ng/mg	uGGT /uCr U/g
Dog (random effect)	0.02	0.02	0.002	0.01	0.003
Age	NS	0.0001	< 0.0001	0.02	0.02
Weight	NS	NS	0.005*	NS	NS
Sex	NS	NS	NS	NS	0.04**
Time point after bite	0.005	< 0.0001	0.0001	< 0.001	0.003

*inverse i.e uMCP-1/uCr decreases with increasing weight

** lower in females

*NS = not- significant.*

#### Urinary biomarker correlations with SSS

In the envenomated dogs, SSS at presentation showed a strong positive correlation with peak uGGT/uCr (Spearman’s ρ 0.62, *P* = 0.0025) and peak SSS was moderately correlated with peak uMCP/uCr (Spearman’s ρ 0.43, *P* = 0.02). No other significant correlations were found between peak SSS or SSS at presentation and peak urinary biomarker/creatinine ratios.

## Discussion

Results from this study are suggestive of mild transient non-azotaemic AKI in dogs envenomated by *V. berus*. These findings are supported by previous studies of snake-bitten dogs [5, 6].

Increased urinary AKI biomarkers have previously been reported in dogs envenomated by snakes compared to healthy controls [6]. Although significant differences in uAlb/uCr between cases and controls existed in this study, the ratios are lower than reported in previous studies [6], possibly due to a milder kidney insult following *V.berus* envenomation compared to other snake species, although an effect of the extra freeze-thaw cycle these samples were subjected to, cannot be ruled out [34]. Urinary albumin can reflect either glomerular or tubular injury [35] but lacks specificity, with increases also seen with extreme exercise [36], macroscopic hematuria and urinary tract infection [37]. Hematuria was detected in several samples in this study but did not exceed 100 RBC/HPF and was not observed to be macroscopic in any sample. We therefore consider hematuria unlikely to have influenced uAlb/uCr ratios in these individuals.

One other study has specifically examined urinary markers for AKI in *V.berus* envenomated dogs, and reported higher uGGT/uCr and uALP/uCr compared to controls, suggestive of renal injury [5]. Urinary GGT/uCr ratios in both cases and controls in our study were in accordance with the aforementioned study, but uALP/uCr was unexpectedly low. Given that ALP and GGT are both renal brush border enzymes, simultaneous leakage into urine upon tubular cell injury is expected. There are several possible explanations for a lack of increase in uALP in our study compared to others. Poor assay sensitivity might be considered most likely, but differences in timing and storage of samples are also possible explanations [38]. An optimal uALP detection window of less than 12 h after renal insult is reported in humans [39]; thus, the detection window may have been missed in our study. Urinary ALP can also originate from the epididymis and prostatic fluid, thus, differences in proportions of intact male dogs between studies might also influence results [40].

Urinary CysC was not significantly different between envenomated dogs and controls in this study. Urinary CysC increases as a result of decreased reabsorption after proximal tubular injury [41], and an association between uCysC concentration and severity of AKI has been described in humans and dogs [42, 43]. Mild AKI is therefore a likely explanation for the lack of increase in uCysC/uCr in envenomated dogs in the present study. A recent study indicates that the assay used in our study might measure lower concentrations of uCysC compared to other assays [44]. Care should therefore be taken in comparing results from different assays.

Urinary KIM-1 is an early and highly sensitive and specific diagnostic biomarker for proximal tubular injury, approved by the US Food and Drug Administration (FDA) as a marker for drug induced AKI in rodent models [45, 46]. Human studies describe upregulated uKIM-1 expression as early as 2 h, and lasting up until 48 h, after toxic or ischemic insult to the kidney [46]. In our study, significant differences in uKIM-1/uCr between envenomated dogs and controls were not observed until T4. Species differences, sample size, and different mechanisms of renal toxicity are possible explanations for the seemingly later induction of uKIM-1 in our study. However, it has also been suggested that uKIM-1 may not be as sensitive and early a marker of AKI in dogs compared to humans [47, 48]. Urinary uKIM-1/uCr and absolute uKIM-1 concentrations in healthy dogs in our study were lower than reported previously, also in one study using the same assay [33, 49]. The same studies reported comparatively higher uKIM-1/uCr and absolute uKIM-1 in dogs with AKI than found in the envenomated dogs in our study. This indicates that although uKIM-1/uCr was significantly higher at T4 compared to both controls and T5, these ratios are overall low and likely represent mild AKI given previous findings of correlations between KIM-1 and extent of injury [50]. All dogs in the present study had uKIM-1/uCr values below the cutoff of 0.75 ng/mg for diagnosing AKI proposed by one canine study [49], further supporting the theory that dogs bitten by *V. berus* might sustain a milder form of renal injury compared to AKI of other etiologies. A recent study suggests that uKIM-1 concentrations measured in the multiplex assay used in this study could be lower than those detected using other assays [44]. An assay specific reference interval is needed and direct comparisons between concentrations detected in different studies should therefore be made with caution.

Neutrophil gelatinase-associated lipocalin is a ubiquitous LMW epithelial protein, subject to glomerular filtration and tubular reabsorption. Local NGAL production is induced in the distal tubule during renal injury [51]. Urinary NGAL may therefore result from either proximal or distal tubular injury. Higher uNGAL/uCr ratios have previously been reported in snake-bitten dogs that developed AKI compared to controls [52], but this is the first study describing increases in uNGAL in dogs envenomated specifically by *V.berus*. In accordance with another canine AKI study [30], uNGAL/uCr ratios were higher already from 12 h after bite in envenomated dogs, compared to controls, indicating its potential use as an early marker of AKI, as has previously been suggested [53]. A recent study indicated that systemic inflammation has a significant impact on uNGAL/uCr ratios [30]. Two forms of NGAL exist, of which the monomeric form is kidney specific, whereas increases in dimeric NGAL are seen in UTI and other inflammatory disease [54, 55]. Assays for detection of monomeric NGAL in canine urine are not currently available, thus the possible lack of specificity should be taken into consideration when interpreting results.

Upregulation of the inflammatory cytokines MCP-1 and IL-8 is described during renal injury in dogs [27, 33] and in human snake envenomation [56]. Few studies have measured uMCP-1 in dogs; hence, less is known regarding its kinetics. The finding of increased uMCP/uCr 12–36 h after envenomation compared to controls in our study, corresponds with previous nephrotoxicity studies [28, 33], indicating that MCP-1 might be useful as an early marker of AKI. An interesting finding in the present study was the inverse relationship between bodyweight and uMCP-1/uCr in envenomated dogs. Although a lower relative venom concentration in larger dogs is a possible explanation, this was considered more likely to be an incidental finding, since a similar effect was not observed for the other biomarkers. A high number of samples in this study had uIL-8 concentrations under the LLOQ. This was also described in another canine AKI model, despite elevations in other AKI markers [33], raising questions as to the sensitivity of uIL-8 as a marker for canine AKI.

The chemokine OPN has rarely been quantified in canine urine, thereby limiting comparisons with our study. Urinary OPN is a sensitive marker for renal tubular injury in rodent drug induced AKI models [57]. Absolute uOPN concentrations in the present study were comparable to those in a canine hemorrhagic shock model [33]. In the aforementioned study, absolute uOPN values did not increase significantly from baseline after induction and treatment of shock, whereas in our study, uOPN/uCr was significantly higher in envenomated dogs 24 and 36 h after bite, compared to controls. The difference in findings might be explained by difference in sample size, mechanism of AKI, or a lack or normalization to creatinine in the other study. A lack of specificity in the presence of inflammation is also possible and thus a systemic contribution to the uOPN measured, cannot be ruled out.

Serum concentrations of MCP-1, IL-8 and OPN can increase in various inflammatory states, including muscle injury and snake bite [57–60]. Given their LMW, we cannot rule out a systemic contribution via glomerular filtration during systemic inflammation induced by snake envenomation [61, 62], and further studies are therefore needed to ascertain the specificity of these markers.

Except for uCysC, all urinary biomarker/Cr ratios were higher 36 h after bite compared to controls. Whilst clearance kinetics of the various biomarkers may differ, our findings suggest that AKI might occur until at least 36 h after *V. berus* bite. A resolution of AKI by 14 days is suggested by a lack of significant difference between cases and controls at T5, but further studies are needed to clarify whether AKI is present beyond 36 h after bite. Although our study design does not allow us to establish a direct benefit of IVFT in the treatment of AKI in these dogs, hospitalization with monitoring and targeted IVFT is a sensible recommendation for dogs envenomated by *V.berus,* and results from our study indicate that this treatment should be implemented for a minimum of 36 h.

In accordance with previous studies [9, 63], sCr concentrations were within the reference interval for all dogs in this study, likely due to a lack of kidney dysfunction, although an effect of hemodilution due to IVFT cannot be ruled out. The increases in SDMA observed in two individuals at T5 were marginal and given that the two dogs also had high USG values at T5, we consider kidney dysfunction unlikely. According to veterinary AKI grading guidelines [16], two of the envenomated dogs in this study would be classified as having AKI grade I due to a non-azotaemic increase in sCr of 26.4 μmol/L within 48 h, and eight dogs due to microalbuminuria at one time point or more. A grading system incorporating novel renal injury biomarkers such as those measured in this study, to identify non-azotaemic AKI biomarker-positive individuals, would be of benefit by allowing early identification and treatment. More work is needed to establish the specificity of many of these biomarkers as well as to generate reference intervals for their clinical use.

In accordance with one other study [5], a positive correlation was found between severity of clinical signs at presentation and uGGT/uCr in our study. Although SSS at presentation might thus be an indicator of peak uGGT/uCr after snake bite, further studies are needed to fully assess its use in this setting, especially since the relationship between uGGT/uCr and extent of injury is unknown. The lack of correlation of SSS with the other biomarkers in this study, suggests that severity of clinical signs after envenomation is of overall limited use in predicting which dogs are most likely to develop AKI.

There are limitations to this study. As previously mentioned, the specificity of uOPN, uNGAL, uIL-8, and uMCP-1 in the face of systemic inflammation needs clarification. However, the parallel increases in the urinary biomarkers evaluated in this study are suggestive of renal tubular injury in dogs envenomated by *V. berus*. Sample size limits the statistical inferences that can be made. Differences in assay sensitivity and imputation methods used for values under LLOQ hinder direct comparisons between our study and others for some of the biomarkers. A high number of samples had values under the LLOQ for uALP, uIL-8 and uAlb, thereby limiting their interpretation. Evidence suggests that uALb/uCr and uNGAL/uCr values are unlikely to be significantly affected by microscopic haematuria and haemoglobinuria, respectively [52]. Likewise, haematuria and haemoglobinuria are unlikely to significantly affect uGGT/uCr [37, 38], but it is unknown to what extent results for the other biomarkers in this study might be affected. Although the extent to which myoglobinuria might interfere with biomarker measurement is unknown, it is thought unlikely to influence the overall conclusions of this study since this was observed only in one dog. Urine samples were not subjected to bacterial culture, and thus subclinical bacteriuria may have been missed, although the extent to which this might influence results is unknown. Treatment with antivenom is a confounding factor, although unavoidable due to the non-interventional nature of this study. Differences in venom dose and individual susceptibly to venom are also factors that might impact our results. Hypotension was not apparent after envenomation in the 23 dogs for which data was available. Two dogs were at moderate risk of TOD due to persistent SAP values > 160 mmHg and we therefore cannot rule out that hypertension, albeit likely situational, may have contributed to proteinuria in these dogs [64]. Renal histopathology would have been a useful addition to this study but was not included due to ethical considerations. Finally, potential glomerular injury was not fully assessed in this study and further studies examining specific markers of glomerular injury in envenomated dogs would therefore be useful.

## Conclusions

Increases in the urinary AKI biomarkers KIM-1, GGT, NGAL, IL-8, OPN, MCP-1 and albumin were indicative of renal tubular injury in dogs 12–36 h after envenomation by *V.berus* in this study, although further work is needed to ascertain the specificity of IL-8, NGAL, OPN and MCP-1. Serum creatinine and SDMA were of limited diagnostic use in this study. Overall, severity of clinical signs did not appear to be a useful indicator of urinary AKI biomarker/uCr ratios, suggesting that AKI can occur regardless of initial clinical assessment. The finding of increased AKI biomarkers 36 h after bite suggests that hospitalisation and supportive treatment of *V.berus* envenomated dogs is sensible.

## Methods

### Animals

Forty-one dogs presenting with a *V. berus* bite to the first opinion emergency service at the small animal hospitals at NMBU, EOD, ADO and AJD between April and October 2018, were evaluated for enrollment to the study. Diagnosis of snake bite and thus inclusion in the study was based on history and presence of consistent clinical signs at presentation (fang marks, local or systemic signs of envenomation). Six dogs were excluded for the following reasons: treatment with non-steroidal ant-inflammatory drugs (*n* = 1), presentation more than 24 h after snakebite (*n* = 2), dry bite (lack of clinical signs within 12 h of the bite) (n = 1), sampling occurring outside of the stipulated time frame (n = 1) and a lack of urine samples (n = 1). Permitted pre-existing diagnoses and treatments included hypothyroidism treated with levothyroxine (n = 1) and mitral valve insufficiency treated with pimobendan (n = 1).

### Physical examination, urine and blood examination

All envenomated dogs underwent physical examination and blood sampling at the following time points post bite: T1 (presentation: 1–7.5 h), T2: 12 (± 2) hours, T3: 24 (± 2) hours, T4: 36 (± 2) hours and T5: 10–23 days. Urine samples were obtained at all timepoints except T1. Treatment decisions were made by the attending clinician.

Midstream voided urine samples were collected at each timepoint following cleaning of the vulva or prepuce with 0.3% chlorhexidine wipes (ICF, Palazzo Pignano, Italy). Macroscopic examination, dipstick analysis (IDEXX UA* strips, IDEXX Laboratories, Inc., Westbrook, Maine 04092) and specific gravity, measured by refractometry, were performed before centrifugation at 450 g for seven minutes. Supernatant was aliquoted into cryotubes and frozen at − 80 °C within 15 min. For all samples, sediment was resuspended in approximately 1 ml of supernatant and immediately examined in unstained and stained (BD Clay Adams Sedi-Stain, Sparks, MD 21152 USA) wet slides. Where findings were made on wet slides, dry preparations were prepared (Hemacolor, Merck KgaA, 64,271 Darmstadt, Germany) and reviewed by two veterinarians. Where differences in cell and cylinder counts existed between veterinarians, the mean of the two observations was used.

Urine supernatant was aliquoted and stored at − 80 °C for a maximum of 9 months prior to ALP and GGT analysis and 17 months prior to multiplex immunoassay analysis of IL-8, OPN, KIM-1, NGAL, CysC, MCP-1 and Albumin. Samples were transported on dry ice to either the reference laboratory at NMBU: Sentrallaboratoriet (SL) (ALP, GGT and creatinine) or to a research laboratory at NMBU for immunoassay analysis.

Blood was collected at T1-T5 through a venous catheter in the cephalic or saphenous vein or using a 21-gauge needle with butterfly extension in the jugular vein into serum tubes and centrifuged at 1100 x g for 10 min, 30–60 min post sampling. Serum was pipetted into cryotubes and stored at − 80 °C for a maximum of 12 months prior to transportation on dry ice to a research laboratory (IDEXX Laboratories, Inc., Westbrook, Maine 04092) and a maximum of 20 months before sCr and SDMA analysis.

### Blood pressure measurement

Indirect blood pressure (Cardell®, Midmark, Versailles, USA) measurements were recorded at T1-T5. A cuff size of approximately 40% limb circumference was placed on either the distal radius or metatarsus with the patient in lateral recumbency. Twelve serial measurements of SAP, DAP and MAP were recorded per time point. Mean SAP, DAP and MAP were calculated after discarding the first two measurements and any obvious outlying values.

### Snakebite severity score (SSS)

Envenomated dogs were assigned a 16-point snakebite severity score (SSS) at each timepoint, using an adaptation of a previously described grading system [65]. Grading criteria are described in Table 3*.*

**Table 3:** 16 -point snake bite severity score grading criteria

	Score
**Respiratory system**	
• Signs within normal limits	0
• Minimal: Mild tachypnea, consistent with pain response	1
• Moderate: Marked tachypnea and/or slightly increased respiratory effort	2
• Severe: Extreme tachypnea and or respiratory insufficiency/failure	3
**Cardiovascular system**	
• Signs within normal limits	0
• Minimal: Tachycardia consistent with pain, general weakness, hypertension	1
• Moderate: Tachycardia, hypotension with tarsal pulse still palpable	2
• Severe: Extreme tachycardia, hypotension with non-palpable tarsal pulse or systolic blood pressure < 80mmgHg, arrhythmia or cardiac arrest	3
**Local wound**	
• Signs within normal limits	0
• Mild: Pain, swelling, ecchymosis, erythema limited to bite site	1
• Moderate: Pain, swelling, or ecchymosis involving less than half the extremity and may be spreading slowly	2
• Severe: Pain, swelling, or ecchymosis involving most or all of one extremity and is spreading rapidly	3
• Very severe: Pain, swelling, or ecchymosis extending beyond affected extremity (in case of head: extending to the neck)	4
**Gastrointestinal system**	
• Signs within normal limits	0
• Minimal: Abdominal pain, tenesmus,	1
• Moderate: Vomiting or diarrhoea	2
• Severe: Repetitive vomiting, diarrhea, haematemesis, or haematochezia	3
**Demeanor**	
• Bright, alert, responsive (BAR)	0
• Minimal: Quiet, alert, responsive (QAR)	1
• Moderate: Lethargic	2
• Severe: Extreme lethargy, collapse	3
**Total Score**	/16

### Control dogs

A cohort of 35 privately-owned healthy control dogs not previously bitten by *V. berus* was recruited using stratified sampling by age and weight. A single blood sample was collected into EDTA and serum tubes from the jugular vein using a 21-gauge needle with butterfly extension. Haematology and biochemistry were performed on EDTA blood and one serum aliquot on the day of collection. The remaining serum aliquot was stored and transported as above, for sCr and SDMA analysis. A single urine sample was collected and prepared as for the envenomated dogs. Healthy status was defined as an unremarkable history and physical examination, no history of chronic disease or any illness within the two weeks prior to recruitment, no clinically significant haematology or serum biochemistry abnormalities and an unremarkable urinalysis on voided urine including less than five leucocytes per high power field (HPF). A mild elevation in sCr (150 μmol/L) was permitted in one very muscled dog where urine specific gravity (USG) was 1.043 and SDMA was within the reference interval.

### Laboratory analyses

Urinary ALP, GGT and creatinine were analyzed at SL on an automated biochemistry analyzer (Advia®1800, Siemens, Germany). Urinary ALP and uGGT were measured using a Modified International Federation of Clinical Chemistry and Laboratory Medicine (IFCC) Method [66, 67]. Urinary and serum creatinine were determined by Jaffe’s reaction using picrate at alkaline pH at SL and a research laboratory (IDEXX Laboratories, Inc., Westbrook, Maine 04092), respectively. Serum SDMA concentration was measured using a high-throughput immunoassay (IDEXX Laboratories, Inc., Westbrook, Maine 04092).

Concentrations of uCysC, uKIM-1, uIL-8, uNGAL, uMCP-1 and uOPN were measured using a 6-plex Luminex xMAP® assay (Milliplex® MAP Kit, Canine Kidney Toxicity Expanded Magnetic Bead Panel 1, EMD Millipore Corporation, MA, USA). Urinary albumin was measured as a single analyte in a Luminex xMAP® assay (Milliplex® MAP Kit, Canine Kidney Toxicity Expanded Magnetic Bead Panel 2, EMD Millipore Corporation, MA, USA), after a second freeze-thaw cycle. Both assays were previously validated by the manufacturer for use on canine urine. Samples were analyzed in duplicate and according to the manufacturer’s instructions. Briefly, samples were thawed at room temperature, vortexed and centrifuged at 14000 RCF at 4 °C for 5 min and then diluted 1:2 in assay buffer for CysC, KIM-1, IL-8, NGAL, MCP-1 and OPN, and 1:500 for albumin. The plates were read on a Bio-Rad 200 instrument (Bio-Rad, USA) and data was processed using BioPlex Manager 6.1 software (Bio-Rad, USA). A 5-parameter logistic regression model was used to generate standard curves for each analyte. Standard curve outliers were excluded by the software if situated outside the standard’s acceptable recovery range (70–130%). Sample duplicates were manually excluded where the coefficient of variation (CV) exceeded 40%. The standard curves were optimized by the software before calculating the concentration of each analyte. Values below the lower limit of quantification (LLOQ) were imputed using the LLOQ, including values below the limit of detection (LOD = mean blank + 3 standard deviations) since these were close in value to the LLOQ.

All urinary biomarkers were normalized to urinary creatinine and expressed as ng/mg creatinine except for ALP and GGT that were expressed as U/g creatinine and Albumin expressed as mg/g. Urinary Alb/Cr ratios of 30-300 mg/g were considered to be positive for microalbuminuria [68].

### Statistical analysis

Statistical analysis was performed using commercially available statistical software packages (JMP Pro 14.3.0, SAS Institute Inc., Cary, NC and GraphPad Prism 8.3.1, GraphPad Software LLC, San Diego, CA). Data were visually assessed for normality. Most data were not normally distributed; thus, median and range are reported throughout. Comparisons of urinary biomarkers between the control group and each of the timepoints for the envenomated dogs were performed using a Wilcoxon rank sum test for non-parametric variables and a t-test for parametric variables. Bonferroni correction was applied to adjust for multiple comparisons.

Repeated measurements of urinary AKI biomarkers in envenomated dogs were analysed in a linear mixed model with age, weight and sex as fixed effects and dog as a random effect. Generalised linear model assumptions were checked by assessing the residuals in each model and data was log, square root or cube root- transformed where appropriate. Post hoc Bonferroni correction was applied. For uAlb, uIL-8 and uCysC a satisfactory transformation was not achieved; thus, ratios of these variables were not compared across timepoints for envenomated dogs.

Statistical analysis of albumin, GGT, NGAL, KIM-1 and IL-8 was performed after exclusion of 11 individual urine samples from five envenomated dogs with leucocyte counts > 5/ HPF due to evidence of an effect of pyuria or urinary tract infection on results in previous studies [49, 69–72].

Correlations between peak AKI biomarker/creatinine ratios and between peak AKI biomarkers and SSS at presentation and peak SSS were assessed using Spearman’s rank correlation.

## Supplementary Information

**Additional file 1.** Breed overview for envenomated dogs and healthy control dogs.

## Data Availability

The datasets used during the current study are available from the corresponding author on reasonable request.

## Abbreviations

AKI: acute kidney injury
ADO: Anicura Dyresykehus Oslo
AJD: Anicura Jeløy Dyresykehus
EOD: Evidensia Oslo Dyresykehus
HMW: high molecular weight
HPF: high power field
IFCC: International Federation of Clinical Chemistry and Laboratory Medicine
IRIS: International Renal Interest Society
LLOQ: lower limit of quantification
LMW: low molecular weight
LOD: limit of detection
LOQ: limit of quantification
NMBU: Norwegian University of Life Sciences
SDMA: Symmetric dimethylarginine
sCr: serum creatinine
SSS: snakebite severity score
uAlb: urinary albumin
uALP: urinary alkaline phosphatase
uCr: urine creatinine
uCysC: urinary cystatin C
uGGT: urinary γ- glutamyl transferase
uIL-8: urinary interleukin-8
uKIM-1: urinary kidney injury molecule − 1
uMCP-1: urinary monocyte chemoattractant protein-1
uNGAL: urinary neutrophil gelatinase-associated lipocalin
uOPN: urinary osteopontin
USG: urine specific gravity
