# Supplementary material for: Serial serum creatinine, SDMA and urinary acute kidney injury biomarker measurements in dogs envenomated by the European adder (Vipera berus)
Source: BMC Vet Res. 2021 Apr 12;17:154. doi: 10.1186/s12917-021-02851-8 (PMC8042969; doi:10.1186/s12917-021-02851-8)
Supplement: Supplementary file 1 — Additional file 1. Breed overview for envenomated dogs and healthy control dogs. [file 12917_2021_2851_MOESM1_ESM.docx]

# Additional File 1: Breed overview for envenomated dogs and healthy control dogs.

| Breed | Envenomated Dogs | Control Dogs |
| --- | --- | --- |
| Akita | 1 |  |
| Australian kelpie | 1 |  |
| Bichon frise | 1 |  |
| Bodeguero |  | 1 |
| Border collie | 1 |  |
| Border terrier |  | 1 |
| Boston terrier | 1 |  |
| Boxer |  | 1 |
| Briard |  | 1 |
| Cavalier king Charles spaniel | 1 | 2 |
| Cocker spaniel | 1 | 2 |
| Crossbreed | 8 | 3 |
| Danish-Swedish farm dog |  | 2 |
| English setter | 4 | 3 |
| Finnish lapphund | 1 |  |
| Flat coated retriever | 2 |  |
| Golden retriever |  | 4 |
| Gordon setter | 1 |  |
| Greyhound |  | 1 |
| Irish setter |  | 1 |
| Irish wolfhound |  | 1 |
| Japanese spitz |  | 1 |
| Kleiner Münsterländer | 1 | 1 |
| Labrador retriever | 2 | 3 |
| Malinois | 1 |  |
| Miniature pinscher |  | 1 |
| Miniature poodle | 1 |  |
| Miniature Schnauzer | 1 |  |
| Nova scotia duck tolling retriever | 1 |  |
| Pointer |  | 2 |
| Saluki |  | 1 |
| Samoyed | 1 |  |
| Shetland sheepdog | 1 | 1 |
| Staffordshire bull terrier | 1 | 1 |
| Standard poodle | 1 | 1 |
| Toy poodle | 1 |  |
